# Anatomy and Physiology

**Published:** 1843-01

**Authors:** 


### PART THIRD.

### Selections from the British and Foreign Journals.

---

### ANATOMY AND PHYSIOLOGY.

### *On the Composition of Fibrin.* By M. Bouchardat.

The author of this communication is well known, as the ingenious introducer of the *gluten bread*, which promises to be one of the most important aids in the dietetic treatment of diabetes. He is at present engaged in a series of experiments on the proximate principles of organized bodies; and he has been led by them to entertain some novel views on the nature of fibrin, of which the following is a brief account.

He premises by stating that his experiments have been made upon fibrin separated from the blood by beating it with a stick, and upon the buffy coat of inflammatory blood. When a piece of the buffy coat is boiled in three or four times its weight of water, after having been well washed in cold water, it is reduced to about half its volume, and the water is found to hold in solution a considerable quantity of a gelatinous substance, which is sometimes enough to form a jelly on cooling. That this is true gelatine, without any mixture of albumen, appears from the action of the usual tests. The proportion of gelatine in the blood is extremely variable. In the blood of a person in health, it is sometimes difficult to establish clearly the presence of gelatine; but in inflammatory affections of the serous and cellular tissues, the proportion increases very greatly. This is a fact of great pathological interest.

Besides gelatine, two other substances are considered by M. Bouchardat to exist in fibrin. When a piece of fibrin is placed in water containing only 1-2000th part of muriatic acid (a mixture so weak as scarcely to affect litmus paper, or to be perceptibly sour to the taste,) it immediately swells and becomes flocculent; by a prolonged maceration the greater part is dissolved; but there remains a substance which is not attacked by an excess of the solvent, and which appears to M. Bouchardat identical with the basis of the epidermic tissues and of their horny appendages. For this substance he proposes the name of *epidermose.*

The acidulated solution appears to contain a large proportion of an albuminous compound, which is precipitated by an excess of nitric or muriatic acids, and redissolved by a still greater excess; flocculi are also precipitated by heat; and an abundant precipitate is thrown down by a solution of bichloride of mercury, prussiate of potass, and tannin. When examined with polarized light, it causes a deviation of the rays to the left. Hence this compound is regarded by M. Bouchardat as identical with the albumen of the egg; and he names it *albuminose.*

The acidulated solution, however, probably contains gelatine also; for M. Bouchardat has ascertained by a parallel experiment, that water thus acidulated will slowly dissolve isinglass at a temperature of about 68° Fahr. This last experiment is considered by him as proving that gelatine pre-exists as such in the animal tissues (which has been denied by some); since it is scarcely to be expected that water so feebly acidulated could effect any considerable transformation at the ordinary temperature.

This solvent action is not confined to muriatic acid, but is possessed by several other acids, even by those which, in larger proportion, precipitate albumen from its solutions, by forming with it insoluble compounds.

The gluten of wheat, when acted upon by a similar acidulated fluid, forms a limpid solution, which resembles, in all its chemical reactions, that of the albuminose of fibrin; but it contains no principle analogous to epidermose. The serum of the blood, the albumen of the egg, and the caseum of milk, when treated in the same manner, afford analogous results.

The following conclusions are drawn by M. Bouchardat from these important experiments:

"1st. Fibrin, when separated from fatty matter, is composed of three proximate principles in variable proportions;—a substance identical with pure non-coagulated albumen, for which I propose the name of *albuminose:* this fluid albumen is imprisoned in a network of a tissue composed of *gelatine*, and of a principle possessing all the properties of the epidermic formation, for which, on this account, I propose the name of *epidermose.*

"2d. The fundamental principle which we find in the albumen of the egg, in the serum of the blood, in the gluten of the cerealia, and in the caseum of milk, is always identical; it is albuminose mixed or combined, sometimes with earthy matter, as phosphate of lime and magnesia; sometimes with alkaline salts; sometimes with fatty matters; which extraneous substances mask its essential properties. When, by the operation of an almost unappreciable proportion of acid, we have destroyed this transient combination, the solution of albuminose possesses identical properties, exerting precisely the same chemical reactions, having the same influence on polarized light, and possessing an energy (other things being equal,) exactly proportionate to the quantity of the substance dissolved."

This last statement may, we believe, be accepted as truth; since it bears a very close correspondence with the results of the analogous experiments of Mulden and other German analytical chemists. And with respect to the former, we do not see any ground for hesitating to accept the conclusion of M. Bouchardat, that gelatine exists in small quantity in the blood; and that its amount is much increased in certain pathological conditions. That gelatine exists in the blood has always been considered probable; especially since our increased knowledge of the chemical relations subsisting between gelatine, fibrin, albumen, &c., has made it almost certain that the gelatine derived from the food cannot be converted into an albuminous or fibrinous tissue, but can only be employed for the nutrition of the gelatinous tissues. (See vol. XIV. p. 510.) That a substance resembling the horny matter of the epidermic tissues, also, should exist in the blood, appears quite consistent with our previous views; since the elaboration of this matter, like that of fat, may be regarded as holding a middle place between the functions of nutrition and secretion, and as probably taking place in the blood during its circulation. By a reference to the comparative table of the constitution of the different proximate principles, which we extracted in our last volume (p. 513,) from Liebig's work, it will be seen that the epidermic substance does not depart so widely as gelatine from the composition of fibrin, albumen, &c.; so that its elaboration from them may be a very simple process.

We can by no means assent, however, to the view of the nature of fibrin which M. Bouchardat seems desirous of founding upon these experiments,—namely, that in the clot of blood there is a network composed of gelatine and epidermose, imprisoning fluid albuminose. It is to be remembered that most of his experiments on this subject were made on the buffy coat; and these cannot be regarded as affording sufficient ground for such a conclusion in regard to the ordinary crassamentum. We have always held that fibrin differs from albumen more in its *vital* properties than in its purely chemical relations; and we see no reason to alter our opinion. Fibrin we believe to be albumen in process of organization; that process is continually taking place in the living body; and the constant withdrawal of the fibrin for the nutrition of the tissues is compensated by a conversion of fresh albumen into fibrin. The process is seen distinctly in the gradual organization of coagulable lymph; and the ordinary coagulation of the blood appears to us a step in the same process, which does not go further because the blood is not in contact with a living surface. A precisely analogous series of gradations is noticeable in plants, where *gum* answers to the *albumen* of animals; whilst the *fibrin* is represented by the peculiar glutinous substance contained in the elaborated sap, which is evidently the pabulum from which the old tissues are nourished, and the new ones formed. To deny the distinct existence of fibrin, as such, (which M. Bouchardat seems inclined to do,) is just as unphilosophical as it would be to deny the existence of muscular fibre as a distinct substance, because, in its purely chemical relations, it may correspond with the albumen of the egg or the gluten of wheat. Moreover, there is a total absence of proof that anything like the gelatinous or epidermoid tissue described by M. Bouchardat really exists in the blood; on the contrary, we believe it to be yet undetermined whether gelatine is ever organized at all, or whether both it and the epidermic substance are not deposited in cells composed of fibrinous tissue, as fatty matter is well known to be. Further, it is well known (especially through the inquiries of Mr. Gulliver) that the fibrinous clot is hardened by long boiling, and that the fibrous appearance it presents becomes more distinct; precisely the reverse would be the case, if the areolar tissue were composed, even in part, of gelatine. In what relative condition the three principles,—namely, the true fibrin, the gelatine, and the epidermose,—exist in the crassamentum, remains a subject for future inquiry, in which microscopical and chemical investigations must go hand in hand. The great increase of gelatine in the blood, during inflammation of the gelatinous tissues, and the corresponding increase of the colourless corpuscles, naturally suggests the idea that the two phenomena may be in some degree related.

*Comptes Rendues*, 1842.

---

### *Revival after Freezing.*

In the winter of 1828-9 in Ireland, Gaimard found that toads could be completely frozen, so that ice lay in small pieces between their muscles, their bodies became quite hard, stiff, and motionless, broke easily and without any effusion of blood, so that, in short, every trace of life disappeared, and yet in ten or twelve minutes they could be revived by immersing them in very slightly warmed water. If they were too quickly frozen they did not revive.

*Bibliothèque Universelle*, 1840; *and Müller's Archiv*, 1841; *Jahresbericht*, p. iii.

---

### *Effects of a Solar Eclipse on Animals.* By M. ARAGO.

IN his report on the eclipse of July 8th, M. Arago mentions in support of a popular notion which he had always disbelieved, that a friend of his put five healthy and lively linnets in a cage together, and fed them immediately before the eclipse. At the end of it three of them were found dead. Other indications of the alarm it produced were seen in a dog which had been long kept fasting, and which was eating hungrily when the eclipse commenced, but left his food as soon as the darkness set in. A colony of ants which had been working actively, suddenly ceased from their labours at the same moment.

*Gazette Médicale. Août* 27, 1842.

---

### *On the Decussation of Fibres at the base of the Brain.* By M. FOVILLE.

THE author points out, what all admit, that the extent of the decussation of fibres commonly demonstrated between the corpora pyramidalia is not sufficient to account for the completeness of the paralysis of one side of the body when the other side of the brain is impaired. "There is no proportion as to size, between their discussating fibres and their point of origin, the crura cerebri, or their termination, the anterior fasciculi of the spinal cord." He has succeeded however in demonstrating "a decussation at the commencement of the spinal cord, not through an extent of a few lines only, or by only a small number of filaments, but through the whole distance which separates the basis of the crura cerebri from the medulla spinalis properly so-called."

The anterior columns of the cord, when arrived at the medulla oblongata, separate from the middle line to give place to the corpora pyramidalia, and at the level of the tuber ascend and pass at right angles over the uppermost of its arcs. The posterior columns on the other hand pass outwards from the apex of the columns going to the cura cerebelli, and leave exposed the anterior fasciculi, which are then visible behind through the whole length of the floor of the fourth ventricle, and along the aquæductus sylvii to the infundibulum.

The crura cerebri, for their part, form, from above downwards, a section of a cone, of which the fibres, instead of taking a straight direction, are turned spirally, and successively approach the median fissure into which they penetrate and plunge into the inner part of the anterior fasciculi of the medulla spinalis. The fibres from the right crus thus pass into the prolongation of the left anterior fasciculus, and *vice versâ*. Along their course, the crura constantly giving off fibres which pass thus from one side to the other, gradually grow thinner, and at their exit from the tuber they constitute only the base of the anterior pyramids. The same circumstances continuing they become finer and finer till their very apices decussate at the boundary between the medulla spinalis and medulla oblongata. The transference of all the elements of the crura cerebri from one side to the other of the nervous axis is thus completed.

The mode of demonstrating this arrangement is to separate the two lateral portions of the nervous axis from the apex of the calamus to the infundibulum by separating the borders of the median grove. When this is done gently, each half may be seen to furnish an infinity of fibrous fasciculi of various size, which pass across the interlacing with each other.

*Bulletin de l'Académie de Médecine. Août*, 1842.

---

### *New Ganglia of Nerves discovered by* Dr. Remak.

In a short memoir, in *Casper's Wochenschrift* for 9th March, 1839, Dr. Remak mentions that he has discovered in the human subject small ganglia on the filaments of the cardiac nerves as they are ramified on the surface of the heart. These ganglia are very small, but when examined under the microscope the characteristic gray corpuscles placed among the filaments of the nerves leave no doubt as to their nature.

In the *Medicinische Zeitung*, of the 8th January, 1840, Dr. Remak announced that he discovered small ganglia upon the branches of the sympathetic as they enter the lungs.

"The par vagum branches of the bronchial nerves (he says) run, as Reisseisen has already pointed out, along the subdivisions of the bronchial tubes nearly to the surface of the lungs. According to my researches they remain white, contain a disproportionate number of white primitive fibres, and present no ganglionic swellings. On the other hand, those from the bronchial plexus which enter the posterior part of the lungs are gray, and contain a great number of gray or organic fibres. They give off smooth non-ganglionic gray branches which run below the pleura, and are probably distributed in it. The principal portion, however, run upon the bronchi, soon lose themselves in the walls of these tubes, and present on their finer ramifications, as in the cardiac nerves, small ganglia, some of which may be recognized by the naked eye, others only by the microscope. In these ganglia I have also, as in those upon the cardiac nerves, ascertained that there is an increase in the organic fibres." Dr. Remak also states that he has discovered ganglia upon the smaller branches of the *superior laryngeal nerve* in man, in swine, the ox, the sheep, and the horse. A pretty large ganglion is placed on both sides, is very constant and generally symmetrical, on the branch of the superior laryngeal nerve which is distributed upon the epiglottis. He also found ganglia upon the branches of the

*glosso-pharyngeal nerve.* All these ganglia are placed upon the filaments of the sympathetic which join themselves to those cerebro-spinal nerves. He has not been able to detect these ganglia upon the gray branches of the nerves, of the kidney, the spleen, and the liver.

In another short paper, in the *Medicinische Zeitung* for 15th April, 1840, Dr. Remak gives the result of his researches upon the nerves of the uterus and the bladder. He states that from the difficulty of procuring the impregnated uterus in the human species, his observations were made upon the human unimpregnated uterus, and upon the impregnated uterus in the lower animals. The nerves of the unimpregnated uterus in the domestic animals are in relation to the size of that organ of very great fineness, even more so than those of the liver. These uterine nerves are whitish from the preponderating number of primitive tubes (primitivröhren) entering into their formation, and in general are without ganglia. In swine, however, small ganglia are regularly found on the trunks of the uterine nerves on both sides of the organ in the unimpregnated condition, but the further divisions of these nerves present none. During the progress of utero-gestation, as Tiedemann has already observed in the human species, the size of the uterine nerves is remarkable, and they may then, as for example in swine, be readily followed into the cornua, and in sheep to the bottom of the uterus.

"I have suggested on these grounds, and this suggestion is elsewhere mentioned, that the increased size of these nerves during utero-gestation was dependent upon an increase in the growth of the organic nervous fibres. This opinion has now been fully established, for the nerves of the impregnated uterus are gray, and composed of a relatively greater number of organic fibres, which during utero-gestation evidently increase the mass, while the number of primitive fibres which come from the spinal cord continue unchanged. Peripheral small ganglia which the analogy of the heart leads me to expect, I have not hitherto found on these gray fibres."

Dr. Remak also mentions that, on the filaments of the sympathetic distributed on the walls of the bladder he has found small ganglia. He believes that to this class of ganglia ought to be referred the small ganglia described by Müller to exist on the nerves of the penis on their entrance into that organ.

---

*On the Erectility of the Iris.* By Professor GROMELLI, of Modena.

FONTANA'S argument against the erectility of the iris, viz., that the finest and most penetrating injections, thrown into the arteries, even immediately after the death of an animal, never produce any extension of the iris, like what happens to the corpora cavernosa, we find controverted in a recent Italian journal. The substances which Professor Gromelli has found to succeed best for minute injections of the iris, are olive or walnut oil, coloured in various ways. He states that these injections penetrate into the most delicate ramifications, without becoming extravasated, and preserve for a long time the parts impregnated with them. In injecting the dead bodies of infants, Professor G. observed that the iris, previously relaxed, swelled up, and that the pupil, previously much dilated, contracted to the extent of more than half its diameter, just as it is seen to do when the retina is struck by the light during life. This fact seems to prove, that the iris is composed of blood-vessels. By the aid of the microscope they are seen disposed, between the ciliary and pupillary edges of the membrane, in rays, partly rectilineal, partly serpentine, while a few run in a circular direction. It results from such a disposition of radiating vessels, fixed at the great circumference of the iris and free towards the pupil, that the sanguineous turgescence expands this membrane and contracts the pupil, while the return of the blood allows the membrane to shrink and the pupil to expand. The professor concludes, that the iris is composed of a vascular turgescible, or erectile, tissue.

*Memoriali della Medicina Contemporanea.*

*On the Pus-like Globules of the Blood.* By Mr. Gulliver.

The pus-like globules found in the blood of patients affected with severe inflammatory and suppurative diseases are very like the pale globules of healthy blood; but there are some points of difference which the author now describes, with the aid of engravings.

In inflammatory affections, the pus-like globules of the blood are generally rather larger, more irregular in size and form, and often more opaque, than the pale globules of the blood of healthy animals. In disease, the pus-like globules are frequently clustered together, and commonly much more numerous than in health. In a fatal case of inflammation and suppuration of the leg, for instance, there was about half as many pus-like globules in the blood as red discs. When the pus-like globules occurring in disease are treated with dilute acetic acid, the molecules composing the nucleus are observed to be surrounded, and often widely separated, by a quantity of very minutely granular matter, which is either less obvious, or even absent, in the pale globules of healthy blood which have been subjected to the action of the same acid.

The globules occurring in disease are sometimes still more peculiar. These, in a fatal case of inflammation and suppuration, were abundant in the venous blood. They are described and figured as composed of one or more red corpuscles, apparently blood discs, included in a delicate and pale envelope.

*London and Edinburgh Philosophical Magazine. Sept.* 1842.

---

*On the Structure of Fibrin, and of False Membranes. Origin of Fibre.* By Mr. Gulliver.

A clot of fibrin is made up of fibrils of extreme delicacy and tenuity, of corpuscles having the characters of primary cells, and of very minute molecules. The fibrils may either form a network, or they may have a parallel arrangement as in perfect membrane. It has commonly been supposed that fibrin only presents an organized appearance when it has coagulated in contact with the living textures, but the fibrils and corpuscles are perfectly distinct in fibrin which has coagulated after its removal from the body.

If fibrin, which has clotted quite independently of the inflammatory process, presents a curious and complicated structure, and is in fact really organized, it may be easily understood (as noticed in the Appendix to Gerber's Anatomy) that inflammation may be quite unnecessary to the healing of wounds, thus supporting the conclusion to which Dr. Macartney long since arrived by a different train of inquiry.

The structure of false membranes is either analogous to or absolutely identical with that of fibrin above mentioned.

The well-known theory of Dr. Schwann ascribes the origin of all the tissues to the formation, in the first instance, of round cells, and the change of these into the various fibres and other textures. Hence the following questions arise:—How is the origin of the fibrils of fibrin to be reconciled with the theory of Dr. Schwann? What is the proof that these fibrils may not be the primordial fibres of many animal textures, the filamentous and its modifications for example? Mr. Gulliver could see no satisfactory evidence that the fibrils of fibrin are merely changed cells, for these fibrils may be observed so quickly after coagulation, that their production, according to the views of Dr. Schwann, would hardly seem possible. Nor did it seem that the fibrils arose from the interior of the blood-discs, like certain fibres depicted in the last researches of Dr. Barry.

*London and Edinburgh Philosophical Magazine. October*, 1842.

